# A review of the challenges, learnings and future directions of home handheld spirometry in interstitial lung disease

**DOI:** 10.1186/s12931-022-02221-4

**Published:** 2022-11-11

**Authors:** Toby M. Maher, Courtney Schiffman, Michael Kreuter, Catharina C. Moor, Steven D. Nathan, Judit Axmann, Paula Belloni, Monica Bengus, Frank Gilberg, Klaus-Uwe Kirchgaessler, Marlies S. Wijsenbeek

**Affiliations:** 1 National Heart and Lung Institute, Imperial College London, London, UK; 2 Hastings Center for Pulmonary Research and Division of Pulmonary, Critical Care and Sleep Medicine, Keck School of Medicine, University of Southern California, Los Angeles, CA USA; 3 Genentech, Inc., South San Francisco, CA USA; 4 Center for Interstitial and Rare Lung Diseases, Pulmonology, Thoraxklinik, University of Heidelberg, Heidelberg, Germany; 5 German Center for Lung Research, Heidelberg, Germany; 6 Center for Interstitial Lung Diseases and Sarcoidosis, Department of Respiratory Medicine, Erasmus MC, University Medical Center Rotterdam, 3015 GD Rotterdam, The Netherlands; 7 The Advanced Lung Disease and Transplant Program, Inova Fairfax Hospital, Falls Church, VA USA; 8 F. Hoffmann-La Roche, Ltd., Basel, Switzerland

**Keywords:** Home handheld spirometry, Idiopathic pulmonary fibrosis, Interstitial lung disease, Remote monitoring, Practical challenges, Technical challenges, Analytical challenges

## Abstract

**Background:**

Patients with interstitial lung disease (ILD) require regular physician visits and referral to specialist ILD clinics. Difficulties or delays in accessing care can limit opportunities to monitor disease trajectory and response to treatment, and the COVID-19 pandemic has added to these challenges. Therefore, home monitoring technologies, such as home handheld spirometry, have gained increased attention as they may help to improve access to care for patients with ILD. However, while several studies have shown that home handheld spirometry in ILD is acceptable for most patients, data from clinical trials are not sufficiently robust to support its use as a primary endpoint. This review discusses the challenges that were encountered with handheld spirometry across three recent ILD studies, which included home spirometry as a primary endpoint, and highlights where further optimisation and research into home handheld spirometry in ILD is required.

**Abstract body:**

Rate of decline in forced vital capacity (FVC) as measured by daily home handheld spirometry versus site spirometry was of primary interest in three recently completed studies: STARLINER (NCT03261037), STARMAP and a Phase II study of pirfenidone in progressive fibrosing unclassifiable ILD (NCT03099187). Unanticipated practical and technical issues led to problems with estimating FVC decline. In all three studies, cross-sectional correlations for home handheld versus site spirometry were strong/moderate at baseline and later timepoints, but longitudinal correlations were weak. Other issues observed with the home handheld spirometry data included: high within-patient variability in home handheld FVC measurements; implausible longitudinal patterns in the home handheld spirometry data that were not reflected in site spirometry; and extreme estimated rates of FVC change.

**Conclusions:**

Home handheld spirometry in ILD requires further optimisation and research to ensure accurate and reliable FVC measurements before it can be used as an endpoint in clinical trials. Refresher training, automated alerts of problems and FVC changes, and patient support could help to overcome some practical issues. Despite the challenges, there is value in incorporating home handheld spirometry into clinical practice, and the COVID-19 pandemic has highlighted the potential for home monitoring technologies to help improve access to care for patients with ILD.

## Introduction

Interstitial lung diseases (ILD) are a diverse and heterogeneous group of respiratory diseases that require regular physician visits and referral to specialist ILD clinics to monitor disease trajectory and response to treatment [1, 2]. However, some patients may experience difficulties or delays in accessing specialist ILD clinics [3–6]. The COVID-19 pandemic has added to the existing challenges associated with accessing care and may have resulted in diagnostic delays and limited the opportunity to monitor disease course and treatment response [7].

In recent years, home monitoring in ILD, specifically handheld spirometry for measurement of forced vital capacity (FVC), has gained attention in clinical practice and trials due to its potential to increase the frequency of data collection, reduce the number of clinic visits required and improve access to care for many patients with ILD [8–10]. As such, home monitoring has received particular interest during the COVID-19 pandemic [7, 8]. Technological developments with smart handheld spirometers allow for real-time data transfer, enabling patients to view their own data via a tablet or smartphone application and physicians to view their patients’ real-time data via a digital platform [11–14]. Further to this, several studies have provided important insights into home handheld spirometry in ILD, reporting that this approach is generally feasible; however, it is not robust enough to be relied upon as an endpoint in clinical trials [9–20].

In this review, however, we discuss the challenges encountered during studies of home handheld spirometry in ILD, along with learnings and future directions. We focus on three recently completed studies where rate of decline in FVC as measured by home handheld spirometry was of primary interest: STARLINER, STARMAP and a Phase II study of pirfenidone in progressive fibrosing unclassifiable ILD (uILD; hereafter referred to as the pirfenidone in uILD study) [15–17].

We describe the practical and technical challenges that were encountered during these studies that affected the quality, and subsequent analysis, of the resulting home handheld spirometry data. Additionally, we highlight areas where further optimisation and research is required to help address the challenges observed with home handheld spirometry in ILD.

### STARLINER

**Fig. 1:**
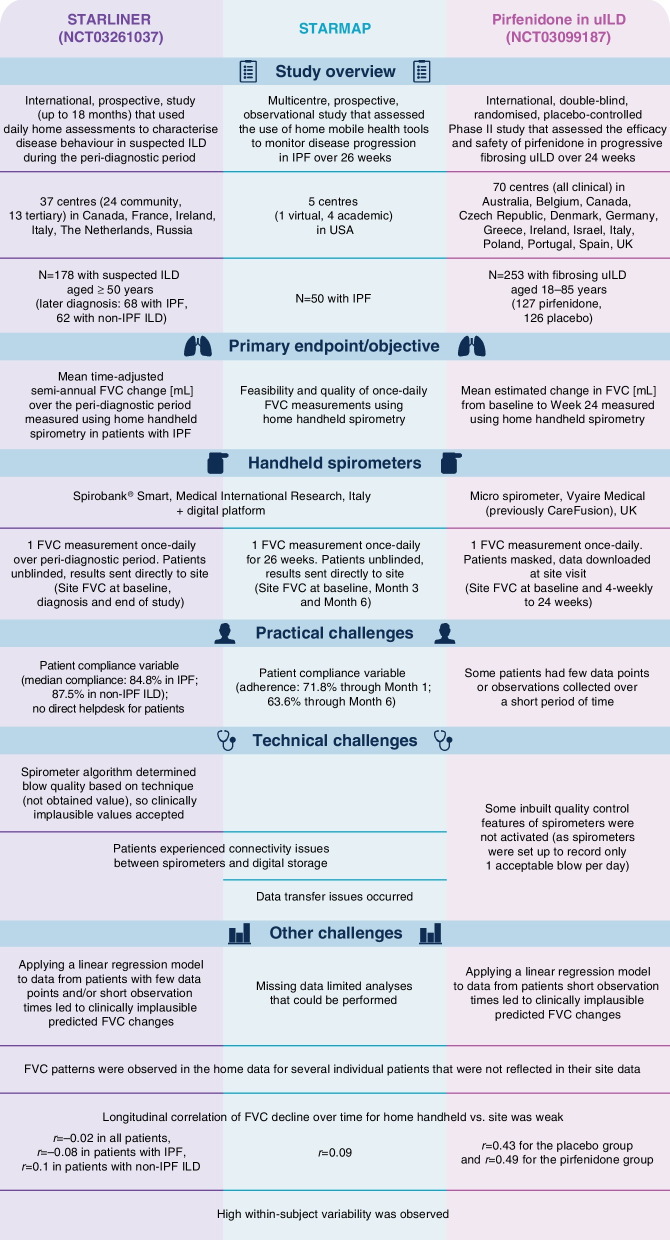
Overview of the STARLINER, STARMAP and the pirfenidone in uILD studies. *FVC* forced vital capacity, *ILD* interstitial lung disease, *IPF* idiopathic pulmonary fibrosis, *uILD* unclassifiable interstitial lung disease

STARLINER (NCT03261037) was an international, prospective study that aimed to use daily home assessments to characterise disease behaviour during the peri-diagnostic period in patients with suspected ILD [17, 21] (Fig. 1). The study length was up to 18 months, with maximum allowed times of 12 months from enrolment to diagnosis of ILD and 6 months from diagnosis to initiating treatment for ILD. A total of 178 patients aged ≥ 50 years with suspected ILD (defined as radiological evidence of ILD in the presence of unexplained dyspnoea on exertion and/or cough) were enrolled, from a total of 37 centres in six countries. During the study, 68 patients were diagnosed with idiopathic pulmonary fibrosis (IPF) and 62 patients were diagnosed with non-IPF ILD. Patients performed one FVC measurement once daily using a portable handheld turbine Spirobank^®^ Smart spirometer (Medical International Research, Italy). Expiratory manoeuvres were categorised by a spirometer-based algorithm as non-acceptable, acceptable except good (due to starting too early or coughing) or good. Patients who did not achieve a good first blow could repeat the test, with a maximum of two blows per day. Patients received a tablet computer that was linked to a digital collaboration platform which allowed patients and physicians to view real-time data. Site visits were mandatory only at baseline, diagnosis and end of study. The primary endpoint was mean semi-annual change in FVC (mL) over the peri-diagnostic period measured using once-daily, home handheld spirometry in patients with IPF; this outcome was also planned as a secondary endpoint in patients with non-IPF ILD.

Findings from STARLINER showed that home handheld spirometry was feasible for most patients with ILD [17]. However, issues encountered with the home handheld spirometers and subsequent measurements led to problems in analysing the handheld spirometry data and prevented confident conclusions regarding disease behaviour in ILD during the peri-diagnostic period.

#### Practical and technical issues experienced in STARLINER

During STARLINER, patient compliance with once-daily, home FVC measurements using handheld spirometry was variable [17]. The median percentage per patient of days with ≥ 1 home FVC measurement recorded was 84.8% for those with IPF and 87.5% for those with non-IPF ILD. Furthermore, 50.0% and 53.2% of patients with IPF and non-IPF ILD, respectively, had at least one gap in home FVC measurements (defined as ≥ 7 consecutive days of missing measurements). Some patients had few data points or observations collected over a short time period during the study.

Connectivity issues occurred between the patients’ devices and the digital collaboration platform, which sometimes prevented patients from performing a once-daily FVC measurement or data transfer [17]. There was no helpdesk support available to patients directly to help overcome these issues; clinicians had to contact the helpdesk on their patients’ behalf. A further technical issue was that the algorithm within the handheld spirometers determined blow quality based on technique rather than the obtained value, and this allowed clinically implausible FVC values being accepted by the devices as ‘good blows’.

#### Other issues experienced in STARLINER

The pre-specified primary analysis in STARLINER used a linear regression model to estimate the semi-annual FVC change for individual patients with at least three FVC measurements taken with the home handheld spirometers.

The group mean FVC changes per cohort would then be calculated and compared. However, since several patients in STARLINER had few data points or short observation times (due to reasons such as patients receiving a specific diagnosis/initiating treatment and subsequently leaving the study at variable times), some extreme estimates of FVC change were observed. The pre-specified estimation of mean FVC changes was heavily impacted by these extreme values, and, therefore, median group FVC changes were selected post hoc for interpretation of the data. A sensitivity analysis excluding patients with short observation times (< 30 days of observation) improved the statistical analysis strategy but did not solve all problems with the data. This highlights that some issues with home handheld spirometry data may go beyond the existence of extreme estimated FVC change in individual patients, and that missing/variable data in patients with a longer observation time may also have contributed to the issues observed in the data.

**Fig. 2:**
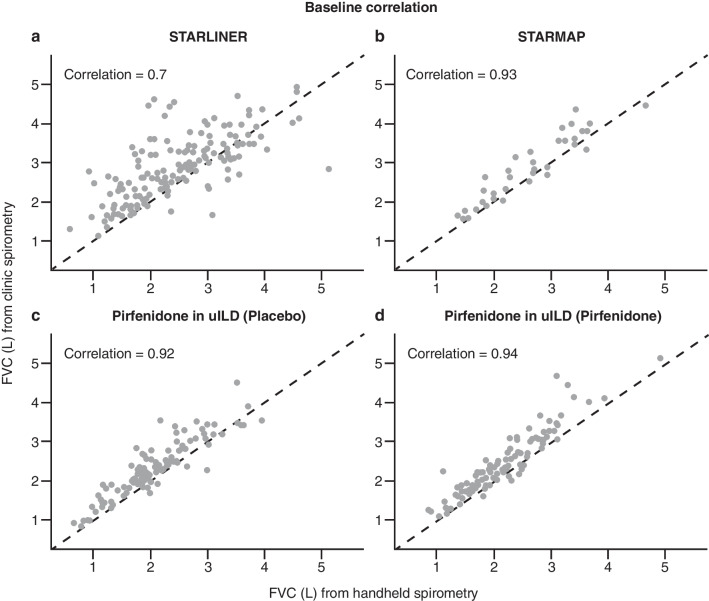
Correlation of FVC at baseline between home handheld spirometry and site spirometry in **a** STARLINER, **b** STARMAP, **c** the pirfenidone in uILD study (placebo group) and **d** the pirfenidone in uILD study (pirfenidone group). For home handheld spirometry, baseline was calculated as the average of assessments collected during the first 7 days. *FVC* forced vital capacity, *L* litres, *uILD* unclassifiable interstitial lung disease

**Fig. 3:**
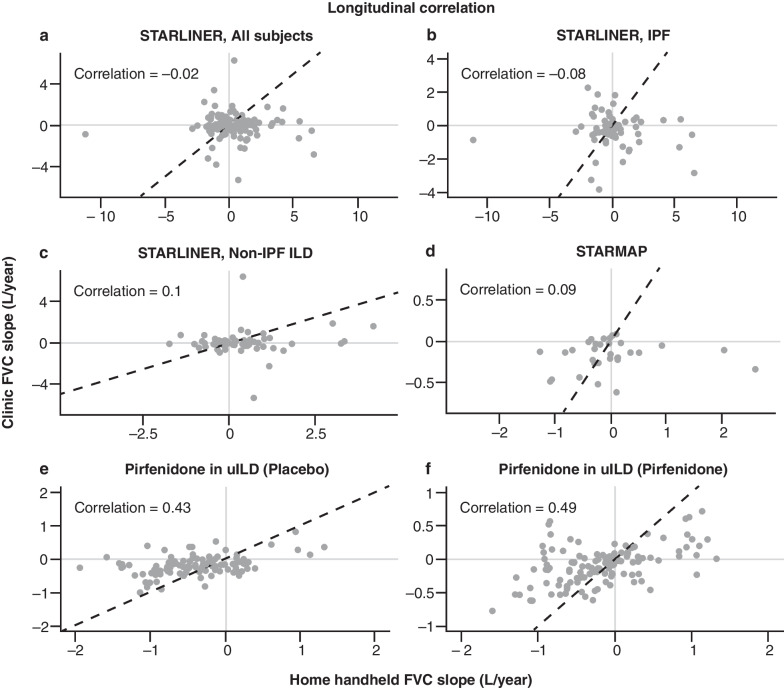
Longitudinal correlation of FVC decline over time between home handheld spirometry and site spirometry in **a** STARLINER, all subjects, **b** STARLINER, IPF, **c** STARLINER, non-IPF ILD, **d** STARMAP, **e** the pirfenidone in uILD study (placebo group) and **f** the pirfenidone in uILD study (pirfenidone group). For home handheld spirometry, baseline was calculated as the average of assessments collected during the first 7 days. *FVC* forced vital capacity, *ILD* interstitial lung disease, *IPF* idiopathic pulmonary fibrosis, *uILD* unclassifiable interstitial lung disease

Of the total 8647 blows performed by patients with IPF and 12,167 blows performed by patients with non-IPF ILD, the handheld spirometer algorithm categorised 54.0% and 60.2%, respectively, as ‘good blows’ and 19.6% and 19.5%, respectively, as ‘acceptable blows except good blows’. FVC measurements obtained using home handheld spirometry (including ‘good blows’ and ‘acceptable except good blows’) and site spirometry at baseline showed a strong/moderate correlation (Pearson’s correlation: *r* = 0.7) for all patients (Fig. 2a). However, longitudinal FVC decline for home handheld spirometry was not correlated with longitudinal FVC decline for site spirometry when looking at all patients enrolled in STARLINER (Pearson’s correlation: *r* = − 0.02), nor when looking at the subgroups of patients with IPF (Pearson’s correlation: *r* = − 0.08) or non-IPF ILD (Pearson’s correlation: *r* = 0.1) (Fig. 3a–c). These findings highlight that strong cross-sectional correlations do not necessarily imply a strong longitudinal correlation (correlation between changes in FVC over time). If between-patient variability is greater than within-patient changes (as is often the case with FVC), then longitudinal correlations can be low even if cross-sectional correlations are high.

**Fig. 4:**
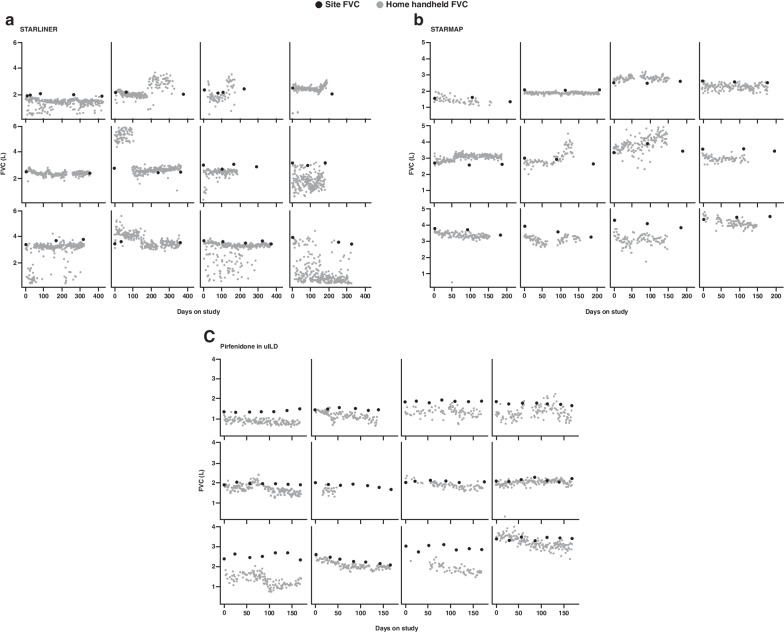
Examples of FVC measurements in individual patients, using home handheld spirometry and site spirometry, in **a** STARLINER, **b** STARMAP and **c** the pirfenidone in uILD study. For home handheld spirometry, baseline was calculated as the average of assessments collected during the first 7 days. *FVC* forced vital capacity, *L* litres, *uILD* unclassifiable interstitial lung disease

It has been hypothesised that home handheld spirometry may lead to more precise treatment effect estimates due to the more frequent measurements. However, the precision of treatment effect estimates depends on within-patient variability in the FVC measurements. The within-patient home FVC measurements from the STARLINER study were considerably more variable than site FVC measurements (variance of site spirometry: 0.050; variance of home handheld spirometry: 0.184; increase in variance: 268%), with some clinically implausible values (examples in Fig. 4a). This highlights that performing more home handheld measurements may not necessarily result in more precise treatment effect estimates.

### STARMAP

STARMAP was a multicentre, prospective, observational study in the USA that assessed the use of home mobile health tools to monitor disease progression over 26 weeks in patients with IPF [16] (Fig. 1). A total of 50 patients aged ≥ 40 years with IPF were enrolled from five centres (one virtual ILD clinic; four academic ILD centres). Patients performed one FVC measurement once-daily using the same type of portable handheld turbine spirometer as in STARLINER (Spirobank^®^ Smart, Medical International Research, Italy). ‘Acceptable blows’ were analysed and data were automatically transferred to a study database (hosted by monARC Bionetworks). Site visits were at baseline, Month 3 and Month 6. The primary objective was to assess the feasibility and quality of once-daily home FVC measurements by handheld spirometry, and several FVC-based endpoints were explored.

#### Practical and technical issues experienced in STARMAP

Similar to STARLINER, compliance with once-daily, home FVC measurements using handheld spirometry in STARMAP was variable [16]. Average compliance, defined as the percentage of days on trial with home handheld FVC data collected averaged across patients, was 71.8% at the end of Month 1 but dropped to 63.6% at the end of Month 6.

Data transfer issues were detected between the home handheld spirometers and cloud-based storage during a 2-month period during the study; however, it is not known if the reduced data collection towards the end of the study was a result of the described technical issues, or if it was due to a change in patient behaviour. Regardless of the cause, this prevented some planned analyses from being performed, such as the estimation of the cross-sectional correlation between home and site FVC measurements at Month 6.

#### Other issues experienced in STARMAP

FVC measurements obtained during STARMAP using home handheld spirometry (using ‘acceptable blows’) and site spirometry were strongly correlated at baseline (Pearson’s correlation: *r* = 0.93; Fig. 2b). However, as observed in STARLINER, high cross-sectional correlations do not necessarily translate into a high longitudinal correlation. In STARMAP, the correlation between FVC decline over the 6-month study period as measured by home handheld spirometry and site spirometry was weak (Pearson’s correlation: *r* = 0.09; Fig. 3d).

Similar to STARLINER, home handheld FVC measurements in STARMAP showed high within-patient day-to-day variability (examples in Fig. 4b). The estimated variance of individual home handheld FVC measurements around the expected value for a given patient was more than double that for site spirometry (variance of site spirometry: 0.017; variance of home handheld spirometry: 0.040; and an increase in variance of 135%). Furthermore, FVC patterns were observed in the home data for several individual patients (e.g., sharp or sudden changes) that were not reflected in their site data (examples in Fig. 4b). If site spirometry measurements were not available for comparison, some of the home handheld spirometry patterns would appear misleadingly real. It is unclear whether these patterns are the result of patients using the devices differently or the result of device error, especially since some of the built-in algorithms to invalidate device errors could not be applied when only one blow was performed.

### Pirfenidone in uILD study

The pirfenidone in uILD study (NCT03099187) was an international, randomised controlled trial (RCT) designed to investigate the efficacy and safety of pirfenidone in patients with progressive, fibrosing uILD [15, 22] (Fig. 1). A total of 253 patients aged 18–85 years were enrolled from a total of 70 clinical centres in 14 countries and were randomly assigned (1:1) to receive 2403 mg oral pirfenidone daily (n = 127) or placebo (n = 126) for 24 weeks. Patients performed one FVC measurement once daily using a portable handheld Micro Spirometer (Vyaire Medical [previously CareFusion], UK). Blows were categorised by a spirometry-based algorithm as 'rejected', 'borderline acceptable' or 'acceptable', and only ‘acceptable blows’ were analysed. Coughing during the manoeuvre rendered a warning message and allowed the patient to perform another blow on the same day. Patients were masked to daily spirometry values. Data were downloaded by site staff at each site visit (every 4 weeks until 24 weeks). The primary endpoint was mean estimated change in FVC (mL) from baseline to Week 24 measured using once-daily, home handheld spirometry.

In the pirfenidone in uILD study, short observation times, high within-subject variability and extreme estimates of FVC change obtained by home handheld spirometry led to problems analysing the home handheld spirometry data [15]. However, mean and median estimated changes in FVC (mL) over 24 weeks using site spirometry (a secondary endpoint) suggested a treatment benefit for pirfenidone over placebo.

#### Practical and technical issues experienced in the pirfenidone in uILD study

Compliance with home handheld spirometry was not a planned endpoint in the pirfenidone in uILD study. Nonetheless, it was observed that some patients had few data points or observations collected over a short time period (e.g., six measurements over 1 week followed by fewer/no measurements in the weeks after) leading to predicted semi-annual decline values that were clinically implausible [15].

A technical issue was that the algorithm within the home handheld spirometers enabled capture of some data that would have been discarded by site spirometry. In particular, blows that were < 6 s but had a flow change of 100 mL in the last 0.5 s were classified as ‘acceptable blows’. The spirometers were also set up to record only one ‘acceptable blow’ per day, with the intent to improve patient compliance and minimise intrusiveness of taking readings. This meant that some inbuilt quality control features that required three blows per day were inactive (e.g., measurement of intra-blow differences of blows done on the same day). These technical issues resulted in undetected day-to-day variability and led to clinically implausible FVC values being accepted by the devices. Overall, home handheld FVC measurements were clinically implausibly low (< 0.5 L) or high (> 6 L) approximately 2.7% of the time.

#### Other issues experienced in the pirfenidone in uILD study

Similar to STARLINER and STARMAP, high within-subject variability and instances of extreme estimated FVC change were observed in some patients in the pirfenidone in uILD study (examples in Fig. 4c). The estimated variance of individual home handheld FVC measurements around the expected value for a given patient was greater than that of site spirometry (variance of site spirometry: 0.018; variance of home handheld spirometry: 0.028; and an increase in variance of 56%).

A linear regression model was to be applied to estimate the FVC change for each individual patient with available FVC measurements taken with the home handheld spirometers, and the mean estimated changes in FVC from baseline to Week 24 for the pirfenidone and placebo treatment groups would then be compared using a Student’s t-test. However, home handheld spirometry measurements collected over a short period of time and clinically implausible values led to a small number of patients having extreme estimates of FVC change that greatly impacted the estimated group means, and the prespecified Student’s t-test comparison was therefore not appropriate.

An updated analysis of the primary endpoint was later performed with the adjudication and elimination of single FVC measurements by home handheld spirometry that were clinically implausible or later deemed unacceptable (data on file). Of the total 42,504 expected home FVC measurements, 32,166 (75.7%) were deemed acceptable in the primary analysis and 25,693 (60.4%) were deemed acceptable in the updated analysis. Whilst the updated analysis eliminated clinically implausible values, it did not eliminate extreme 24-week estimates of FVC change for patients with short observation times. The updated analysis showed no significant treatment difference between groups in mean estimated FVC decline from baseline to Week 24 measured by home handheld spirometry, but a benefit in favour of pirfenidone over placebo in median estimated FVC decline from baseline to Week 24  measured by home handheld spirometry was shown (− 85.6 mL vs. − 183.5 mL, treatment difference 97.8 mL; p = 0.0274). These findings were supported by sensitivity analyses of the updated data based on excluding patients with few data points (< 3 site spirometry measurements) or extreme estimated FVC change from baseline (below − 1000 mL or above + 1000 mL) (data on file). Furthermore, a sensitivity analysis was performed with mean FVC decline estimated by a linear mixed effects model, which could better accommodate missing data.

When this model was applied to the home handheld spirometry data, the treatment benefit in mean estimated FVC decline from baseline to Week 24 for pirfenidone over placebo was similar to that provided by site-based spirometry and reached statistical significance (− 71.7 mL vs. − 184.7 mL, difference 113.0 mL; p = 0.0046).

In the total study population, FVC measurements obtained by home handheld spirometry and site spirometry at baseline were strongly correlated for the placebo and pirfenidone groups (Pearson’s correlation: *r* = 0.92 and *r* = 0.94, respectively; Fig. 2c, d). By contrast, longitudinal correlations of FVC measurements from baseline to Week 24 between the home handheld and site spirometry were moderate/weak for the placebo and pirfenidone groups (Pearson’s correlation: *r* = 0.43 and *r* = 0.49, respectively; Fig. 3e, f). These data further support that high cross-sectional correlations do not necessarily translate into a high longitudinal correlation.

### Integrating challenges and learnings from other ILD studies using home handheld spirometry

In addition to the STARLINER, STARMAP and pirfenidone in uILD studies, other studies in ILD have included home handheld spirometry as part of the study design [9–20]. Of note, in the INMARK RCT investigating the effects of nintedanib on circulating biomarkers in patients with IPF (N = 346), patients performed home handheld spirometry at least once-weekly (ideally daily) over 52 weeks [19]. Additionally, a multicentre RCT in the Netherlands specifically evaluated a home monitoring programme that included once-daily handheld spirometry plus standard of care (SoC) versus SoC outpatient clinic visits only in patients with IPF (N = 90) over 24 weeks [12]. The remaining ILD studies included prospective, observational studies and a pilot study.

#### Optimal frequency of home handheld spirometry measurements

Guidelines for site spirometry published by the American Thoracic Society/European Respiratory Society recommend that, for each FVC measurement, patients perform three good quality blows and that the best of the readings is used [23]. Few ILD studies have extrapolated this recommendation to protocols for home handheld spirometry [9, 18–20], and there are currently no official guidelines for home handheld spirometry.

An alternative to daily spirometry using one FVC measurement might be weekly spirometry using the best of three FVC measurements. In INMARK, mean weekly adherence over 52 weeks was 86%; adherence decreased over time but remained > 75% in each 4-week period [19]. In a prospective, longitudinal cohort study in patients with IPF (N = 25), also using this approach, mean adherence over 24 weeks was 90.5% but decreased over time, especially after 24 weeks [9]. A pilot (N = 10) study in patients with systemic sclerosis-associated ILD found lower within-patient FVC variability with best of three FVC measurements weekly than with one FVC measurement daily (mean coefficient of variation 2.45% vs. 3.86% over 6 weeks) [14]. Overall, more research is needed to determine the optimal schedule for home handheld spirometry; the patient voice will be important when developing best practice guidelines.

#### Patient training, automated alerts, helpdesk support

Across ILD studies using home handheld spirometry, patients received initial training on the use of the home handheld spirometers and related equipment [9–20] and most were also provided ongoing or refresher training [10, 12, 13, 15–19]. However, it should be noted that the training provided in the three ILD studies that were the focus of this review did not prevent the practical and technical challenges encountered with home handheld spirometry, nor the other issues observed with the data. Furthermore, some home handheld spirometers alerted patients if the technique of a blow was not of good quality [15, 17] or to call their physician when appropriate, e.g., for large FVC declines [19].

In some ILD studies with real-time data transfer, an automated email reminder was sent to patients and hospitals when spirometry was not performed or in cases of a significant FVC decline (> 10% predicted) [11, 12]. However, other studies have reported that the inbuilt algorithm within the home handheld spirometer accepted clinically implausible values [15, 17], highlighting potential quality control issues and the need to optimise the algorithms within home handheld spirometers. A helpdesk to support patients with these technical issues (e.g., connection problems) appears to be beneficial when using home handheld spirometers in clinical studies [12].

#### Longitudinal correlations between home handheld spirometry and site spirometry

Across ILD studies, prespecified cross-sectional correlations between home handheld and site FVC values were consistently strong/moderate at baseline, and at later timepoints where available [9, 11, 13, 14, 18–20]. For example, in INMARK, cross-sectional correlations ranged from *r* = 0.72 to *r* = 0.84 [19]. In the RCT of a home-monitoring programme as an add-on to SoC versus SoC alone, cross-sectional correlation values for FVC measurements were *r* = 0.97 at baseline, *r* = 0.97 at 12 weeks and *r* = 0.96 at 24 weeks [12].

However, longitudinal correlations for FVC decline over time were consistently moderate/weak across ILD studies [16, 18, 19], where analysed. For example, in INMARK, longitudinal correlations between home and site spirometry were weak (*r* = 0.26 for rate of FVC decline over 52 weeks) [19]. The highest longitudinal correlations were observed in a RCT of a home-monitoring programme plus SoC versus SoC alone, where the longitudinal correlation for FVC slopes over time was *r* = 0.58 [12]. Within-patient variability in FVC measurements was observed across the ILD studies [9–14, 16, 18, 19]; interestingly, one study identified that patients with progressive disease had higher FVC variability within the first 28 days of home spirometry versus patients with stable disease [18]. Ultimately, these are major challenges that need to be addressed before home handheld spirometry can be truly successful in clinical trials.

### Future directions for home handheld spirometry in ILD

#### Legal and ethical considerations

Prior to widespread implementation of home monitoring, third parties involved with the technology and the data storage must comply with privacy and confidentiality regulations and any other relevant local certification requirements (e.g., CE marking in Europe) [24]. Continuity of care and equal access to care and research for patients who may not find home monitoring suitable for their needs, or do not have the means or skills for online monitoring, are essential.

An area for future research is how a patient’s long-term psychological well-being may be impacted as a consequence of viewing their own real-time data. In surveys, patients with ILD reported that home monitoring (including smart handheld spirometry) helped them to better look after their health [25], that it gave them better insights into their disease course, they felt reassured, and found it pleasant to have an overview of results [12]. A RCT in patients with IPF found that home monitoring plus SoC tended to increase psychological well-being versus SoC alone, as measured using the King’s Brief Interstitial Lung Disease Questionnaire, and did not increase anxiety and depression levels, as measured using the Hospital Anxiety and Depression Scale [12]. Whilst some patients may appreciate the convenience and control, it is not yet clear if other patients may be negatively affected by the intrusion of a medical procedure into their personal time, or from frequent viewing of a longitudinal decline in their FVC measurements. Furthermore, it is not yet known whether providing patients with a more granular understanding of their disease trajectory will impact long-term reporting of health-related quality of life.

#### Implications for clinical trials and real-world registries

Based on data from clinical trials, home handheld spirometry is not ready to replace site spirometry in estimating FVC decline as a primary endpoint in future trials [15–17]. However, in the era of COVID-19, home monitoring may be of value as an exploratory endpoint.

Home monitoring might facilitate international real-world registries, as distances can be bridged online and, as well as handheld spirometry, patients can collect data on other physiological parameters, symptoms and quality of life [26]. The feasibility of a patient-led registry using home monitoring in patients with fibrotic ILDs is currently being investigated (NCT04304898).

Based on the issues observed in STARLINER, STARMAP, pirfenidone in uILD and other ILD studies to date, considerations for future clinical trials and real-world registries that use home handheld spirometry should include how to minimise collection of implausible FVC measurements, how to handle missing data, how to improve longitudinal correlations with site spirometry and how to reduce within-patient variability. Furthermore, additional insights are needed regarding the burden and impact on patients of long-term home spirometry data collection.

#### Implications for clinical practice

Home handheld spirometry can complement site spirometry in clinical practice, potentially reducing the number of outpatient clinic visits required. Advantages have been seen during the COVID-19 pandemic where handheld spirometry and other home monitoring technologies have been key to maintaining quality of care in patients with ILD [7, 8]. With face-to-face interactions between healthcare professionals and patients minimised, many consultations have instead taken place via videoconferencing. Furthermore, in-clinic pulmonary function tests have not been possible as they are aerosol generating procedures, meaning that these have been replaced with home handheld spirometry at some centres.

Aside from a clinical trial setting, home handheld spirometry may be particularly convenient for patients who live some distance from a specialist ILD centre or do not currently have access to specialised care. ILD physicians tend to be positive about the implementation of home monitoring and believe that it can be beneficial in clinical practice and in a research setting [27]. A feasibility trial from the German Center for Lung Research is currently analysing whether home handheld spirometry can detect or predict acute exacerbations early; this could provide a prompt for the patient to have a site FVC measurement for validation. However, the financing of home handheld spirometry in clinical practice will require consideration, as many countries do not reimburse these novel ways of care.

## Conclusions

Across the STARLINER, STARMAP and pirfenidone in uILD studies, similar challenges were experienced with home handheld spirometry. Indeed, in all three studies, patterns observed in the home handheld spirometry measurements were not reflected in site spirometry measurements, and home handheld measurements were more variable than site spirometry measurements. Additionally, despite strong cross-sectional correlations, all three studies reported poor longitudinal correlations between home handheld spirometry and site spirometry. ILD studies that have used home handheld spirometry have also highlighted key challenges associated with the technology and areas for optimisation. Implementing tools such as refresher training, automated alerts of problems and patient support may help to overcome some of these issues in the future. Ultimately, the challenges described in this review highlight that while home handheld spirometry may be a valuable tool in ILD, it requires further optimisation and research before it can be used as part of a primary endpoint in clinical trials.

## Data Availability

Qualified researchers may request access to individual patient-level data through the clinical study data request platform (https://vivli.org/). Further details on Roche's criteria for eligible studies are available here (https://vivli.org/members/ourmembers/). For further details on Roche’s Global Policy on the Sharing of Clinical Study Information and how to request access to related clinical study documents, see here (https://www.roche.com/research_and_development/who_we_are_how_we_work/clinical_trials/our_commitment_to_data_sharing.htm).

## Abbreviations

FVC: Forced vital capacity
ILD: Interstitial lung disease
IPF: Idiopathic pulmonary fibrosis
RCT: Randomised controlled trial
SoC: Standard of care
uILD: Unclassifiable interstitial lung disease
